# Behavioural and social drivers of human papillomavirus vaccination in eThekwini District of KwaZulu-Natal Province, South Africa

**DOI:** 10.1371/journal.pone.0311509

**Published:** 2024-12-31

**Authors:** P. Bhengu, D. Ndwandwe, S. Cooper, P. D. M. C. Katoto, C. S. Wiysonge, M. Shey

**Affiliations:** 1 Faculty of Health Sciences, Department of Medicine & CIDRI-Africa, Institute of Infectious Diseases and Molecular Medicine (IDM), University of Cape Town, Cape Town, South Africa; 2 Cochrane South Africa, South African Medical Research Council, Cape Town, South Africa; 3 Division of Epidemiology and Biostatistics, Centre for Evidence-Based Health Care, Stellenbosch University, Cape Town, South Africa; 4 Centre for Tropical Diseases and Global Health, Catholic University of Bukavu, Bukavu, Democratic Republic of Congo; 5 School of Public Health and Family Medicine, University of Cape Town, Cape Town, South Africa; 6 Vaccine-Preventable Diseases Programme, World Health Organization Regional Office, Brazzaville, Congo; Greenebaum Cancer Center, Institute of Human Virology, University of Maryland School of Medicine, UNITED STATES OF AMERICA

## Abstract

**Background:**

Cervical cancer is the second most common cancer in women in South Africa. Infection with high-risk types of human papillomavirus (HPV) is the cause of cervical cancer, which can be prevented by HPV vaccination. However, there is wide variation in HPV vaccination coverage among the urban districts of South Africa; with the lowest coverage being 40% in eThekwini, KwaZulu-Natal. There could be many factors which affect HPV vaccine uptake in eThekwini District. Thus, this research aims to investigate the behaviourial and social drivers of HPV vaccination in this district.

**Methods:**

The study will consist of two phases. We will apply a convergent parallel mixed methods approach, including a quantitative survey (phase 1) and in-depth interviews (phase 2) among caregivers and frontline healthcare workers to determine the drivers of HPV vaccination uptake.

**Discussion:**

The study will provide knowledge on the main barriers facing HPV vaccination and provide contextually-tailored solutions for how these barriers might be addressed. A policy brief will be formulated from this study aimed at government policymakers and other stakeholders who formulate or influence policy, respectively. In addition, we will disseminate the findings through peer-reviewed publications in scientific journals.

## Introduction

Cervical cancer is the second most common cancer in women in South Africa [1]. Infection with oncogenic types of human papillomavirus (HPV), called high-risk, is the cause of cervical cancer, which can be prevented by HPV vaccination [2]. Other STIs that enhance the risk are herpes, chlamydia, gonorrhea, syphilis, and HIV/AIDS [3]_._ Currently, there are three HPV vaccines in use around the world: a bivalent HPV vaccine, a quadrivalent HPV vaccine, and a nonavalent HPV vaccine [2]. All three vaccines have shown a high degree of safety, immunogenicity, and efficacy against the most oncogenic forms of HPV which are HPV types 16 and 18 [3].

Since 2008, the bivalent and quadrivalent HPV vaccines have been available in the private sector in South Africa [1]. However, a nationwide, public school-based HPV vaccination program was launched in 2014. Girls from grade four, aged nine or older, received two doses of the bivalent HPV vaccine at intervals of six months each [4]. An assessment of the programme’s performance revealed high coverage of more than 80% of eligible adolescents with at least the first dose of the vaccine. However, there was a wide variation in coverage between and within districts; the lowest coverage being 40%, registered in eThekwini District in KwaZulu-Natal Province [3]. There could be many barriers and facilitators affecting the HPV vaccine uptake not only the province of KwaZulu-Natal, but South Africa in general. These include logistics of ensuring access and affordability to the psycho-social factors that influence vaccination-seeking behaviours [2,5–8]. There is thus a need to understand the reason for sub-optimal district HPV vaccination coverage in KwaZulu-Natal, to ensure that evidence informs the design and evaluation of tailored interventions to increase HPV vaccine uptake.

Unless optimal HPV vaccination coverage is equitably attained throughout South Africa, the protective value which HPV vaccines deliver in the country would not be achieved. Characterising the reasons for low HPV vaccination coverage can enable direct comparisons among different factors, and help guide the development, implementation, and monitoring of interventions to improve vaccination. Such work should build on evidence-based research [9] that will guide practitioners in developing successful community and clinical interventions. We will adopt the "Measuring Behavioural and Social Drivers of Vaccination (BeSD)" approach, developed under the auspices of the World Health Organisation (WHO), for this study [10]. The BeSD Working organization, a multidisciplinary organization of experts and immunization partners from throughout the world, was founded by WHO in 2018 to provide tools to comprehend and evaluate behavioral and social determinants of vaccination [5].

**Fig 1:**
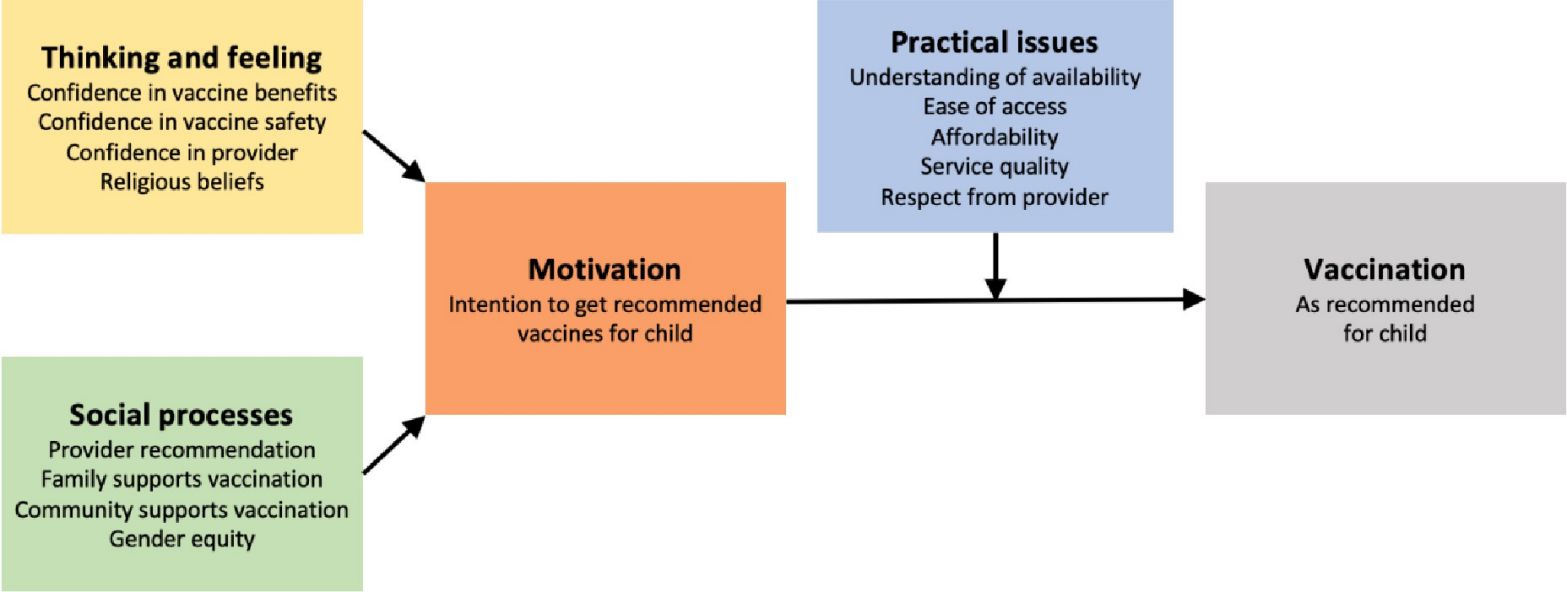
The Behavioural and social drivers of Vaccination Framework (Source: WHO. Understanding the behavioural and social drivers of vaccine uptake. WHO Position Paper-May 2022. Weekly Epidemiological Record 2022;97(20):209–224).

The BeSD tools measure four domains that play a major role in influencing the uptake of vaccines (see Fig 1): The Behavioural and Social Drivers of Vaccination Framework (Source: WHO. Understanding the behavioural and social drivers of vaccine uptake. WHO Position Paper—May 2022. Weekly Epidemiological Record 2022;97(20):209–224.) (1)The thinking and feeling domain, which includes the cognitive and emotional responses towards vaccines. (2) Social processes, which are the social norms and reccomendations on vaccines. (3) Motivation, which includes the willingness and hesitancy towards vaccination. (4) Practical issues, which includes the practical barriers faced when getting vaccinated [10]. Assessing all domains will enable more comprehensive planning and evaluation at national and district levels. The BeSD tools include a quantitative survey and in-depth interview guides and were initially developed for childhood vaccination. We will adapt and validate them for application to HPV vaccination in South Africa (Fig 1): The Behavioural and Social Drivers of Vaccination Framework (Source: WHO. Understanding the behavioural and social drivers of vaccine uptake. WHO Position Paper—May 2022. Weekly Epidemiological Record 2022;97(20):209–224).

### Study objectives

The aim of this research is to (1), investigate the behaviourial and social drivers of sub-optimal HPV vaccination uptake in eThekwini District of KwaZulu-Natal Province in South Africa and (2), to develop contextualised strategies to increase the vaccination coverage.

The study will consist of two phases, each with a specific objective.

The primary objective of Phase 1 is to assess factors associated with the uptake of HPV vaccination from caregivers of children between 9 years and 14 years of age (i.e. the age group recommended for HPV vaccination) in eThekwini District.

The objective of Phase 2 will be to use BeSD qualitative interview guides to obtain contextual data on how caregivers of children and healthcare workers experience and understand the drivers of HPV vaccination.

## Materials and methods

### Study design

The research will employ a convergent parallel mixed-methods approach, integrating both quantitative and qualitative methodologies. The study will adopt a cross-sectional observational design, to be conducted in various communities within the eThekwini District of KwaZulu-Natal Province, South Africa. This design is chosen to effectively capture a snapshot of current attitudes, beliefs, and behaviors related to HPV vaccination among caregivers in a diverse range of urban, suburban, and rural settings. This design includes a survey for quantitative analysis and employs focus group discussions and in-depth interviews for qualitative insights. This mixed method approach ensures a comprehensive understanding of the subject matter, leveraging the strengths of both methodologies: interviews for detailed individual perspectives and focus groups for interactive insights and broader viewpoints. The triangulation of data from these sources will enhance the credibility and dependability of the findings.This phase of the study is characterized by a phenomenological approach, focusing on the lived experiences and perceptions of caregivers and healthcare workers related to HPV vaccination [11]. Phenomenology is selected for its strength in providing deep insights into human experiences, particularly useful in understanding complex behaviors like vaccine uptake. By capturing the essence of participants’ experiences, beliefs, and attitudes towards HPV vaccination, this approach allows the research to delve into the subjective realm, offering a rich, in-depth understanding of the factors influencing vaccination decisions. This qualitative design is particularly adept at uncovering the nuances and complexities that underlie health behaviors, which are often not apparent in quantitative research.

The study will utilize transcendental phenomenology. This methodology focuses on uncovering the fundamental structures of human consciousness and perception, aiming to understand how individuals experience phenomena in their lives and reveal the inherent meanings and essences of these experiences, regardless of specific cultural or historical contexts.

### Study setting

The study will be conducted in eThekwini Municipality, KwaZulu-Natal Province, South Africa. This diverse municipality, home to over 3.5 million people, presents a range of socio-economic, environmental, and political challenges.

### Study population

The study population comprises caregivers of children aged 9 to 14 years and frontline healthcare workers. This demographic selection is crucial given the prevalence of children under 14 (25% of the population) and the significant role of females (over 40% of households are female-led) and education level (61.2% with primary education) in the community [12].

## Study procedures

### Participant recruitment for phases 1 and 2

Recruitment will take place in Chatsworth, Embo, uMlazi, and Wentworth areas. Setting contexts differ in terms of health care service organization, level of resources and area development (Embo being the least developed). Participants will be recruited in schools, communities, and healthcare facilities. We will explain the study aims and rationale during recruitment and during the consent process.

The study will aim for a representative sample to include diverse caregiver demographics in eThekwini District, reflecting the target population. Recognizing the complexities of representative sampling, we will devise a sampling plan guided by best practices in survey methodology. This plan will ensure that caregivers from the same areas as healthcare workers are included. The quantitative survey and in-depth interviews will be conducted concurrently across these areas. Prior to data collection, we will engage with local stakeholders, including service providers and community leaders, to facilitate this process.

### Phase 1: Quantitative approach

By adhering to the STROBE (Strengthening the Reporting of Observational Studies in Epidemiology)guidelines, this study will provide a comprehensive and detailed framework, ensuring methodological rigor and clarity in investigating the drivers of HPV vaccination in the eThekwini District.

### Participants: Inclusion and exclusion criteria

Participants will include caregivers of children aged 9 to 14 years living in the eThekwini District. Inclusion criteria are residency in the district and having at least one child within the specified age range. Exclusion criteria include non-residency in the district and inability to provide informed consent.

### Variables: Primary and secondary

Primary variables of interest include behavioral and social factors such as knowledge about HPV, attitudes towards vaccination, cultural beliefs, and perceived barriers to accessing HPV vaccination. Secondary variables will involve collecting demographic data (age, gender, education level, and socioeconomic status) to facilitate understanding of how these factors may influence vaccination behaviors.

### Data sources/measurement: Questionnaire development and adaptation

The study will utilize a structured questionnaire adapted from existing validated instruments to suit the local context. This involves translating the questionnaire into local languages and making cultural adjustments for relevance. Our focus will be on modifying the BeSD (Behavioral and Social Drivers) questionnaire, which was initially developed for childhood vaccines and has been recently revised by the WHO to assess COVID-19 vaccine willingness, for its application to HPV vaccination [13].

The BeSD framework is designed to explore and measure the various behavioral and social factors that influence vaccination decisions. It encompasses a range of elements including individual beliefs, social norms, motivation, and trust in health services. By assessing these factors, the BeSD model aids public health professionals and researchers in identifying key drivers and barriers to vaccine uptake, thereby facilitating the development of more effective public health strategies and communication efforts.

To ensure the clarity and relevance of the questionnaire, pilot testing will be conducted through cognitive interviews. This will involve two rounds of interviews in one of the study areas, each involving 4 to 8 participants. The aim of this pilot testing is to ensure that participants can easily understand the questions and that their responses accurately reflect the intended constructs. This step is crucial to confirm that all survey items, including the questions and their respective response options, are not only correctly translated but also convey the intended meanings in the context of HPV vaccination.

### Sample size calculation and justification

The planned sample size for our survey, targeting caregivers of children aged 9 to 14 years, is set at 800 participants. This sample size is chosen to adequately assess factors associated with the uptake of HPV vaccination in this age group within the eThekwini District, ensuring statistical power for the primary objective of the study. However, this size also aligns with recommendations for scale validation and is suitable for conducting exploratory factor analyses, ensuring the reliability and validity of the survey scale in capturing the behavioural and social drivers (BeSD) of HPV vaccination which is the secondary outcome of the study [14]. Statistically, this sample size also achieves an 80% power to detect small correlation effects (r = 0.2) [10] with a significance level of 0.05, thereby enabling the identification of even subtle associations within the data. Such a sample size is not only statistically robust for the intended analyses but also adheres to the BeSD framework requirements, ensuring comprehensive coverage of the various factors influencing vaccine uptake.

### Sampling technique

To ensure that the sample accurately reflects the diverse population of the eThekwini District, we will employ a cluster random sampling technique, a method that emphasize its effectiveness in achieving representativeness in diverse populations [11]. Cluster sampling involves dividing the population into clusters or groups, such as geographical areas, and then randomly selecting clusters for inclusion in the study. This method improves data collection efficiency, especially when dealing with large and diverse populations across various geographic locations, and contributes to ensuring proper representation of different areas within the district.This approach is in line with advocacy for capturing a wide range of perspectives in educational and social research [15]. Each stratum will be proportionally represented in the sample, in accordance with the district’s demographic distribution, to ensure a comprehensive understanding of the factors influencing HPV vaccine uptake. Additionally, caregivers will be recruited from varied healthcare access points, including public clinics, private practices, and community health centers. This strategy, helps in understanding different healthcare experiences and attitudes towards HPV vaccination [11]. Furthermore, the sample will include caregivers with different employment statuses and levels of community engagement to provide a nuanced understanding of community-level influences on health behaviors [16].

### Data collection

Eligible individuals will be asked to sign a consent form after research assistants have explained the importance and procedures of the study.A structured questionnaire (Appendix 1 in S1 File) will then be administered by the trained research assistants to collect the required data from consenting participants. Eligible participants will be recruited during their routine visits at clinics and schools and will be asked to answer a validated questionnaire.

### Expected outcomes

The primary outcome of this phase is identifying key factors influencing HPV vaccine uptake among caregivers of children aged 9 to 14 years, wherein the data will be accessed from the caregivers, who are also the ones making the decisions about whether these children get vaccinated or not. This will be quantitatively assessed by measuring the level of awareness and knowledge about HPV. Specifically, the proportion of participants affirmatively responding to questions about their awareness of HPV, its link to cervical cancer, and the availability of the HPV vaccine for grade five girls in public schools, will be calculated as a percentage of the total sample. Additionally, we will assess the willingness to vaccinate daughters or close relatives against HPV. This will involve creating a binary variable, where responses indicating willingness or actual vaccination will be categorized as positive, while undecided and negative responses will be grouped together.

A secondary outcome will be the development and validation of a quantitative tool to measure the behavioral and social drivers of HPV vaccination within the eThekwini district. This involves adapting and validating the BeSD (Behavioral and Social Drivers) tool for HPV vaccination. The significance of this adaptation lies in its potential applicability beyond the immediate study context, extending to other regions in South Africa, various target groups within the country, and potentially to vaccination behavior in general. This effort will mark one of the first instances of validating the BeSD tool specifically for HPV vaccination, thereby making a significant contribution to the field of public health research.

### Addressing potential biases

The study will implement strategies to minimize biases, including selection bias and information bias. These strategies will involve training of researchers, ensuring random sampling, ensuring and maintaining participant confidentiality, which primarily addresses social desirability bias, and standardizing data collection methods with the help of training, protocol development, pilot testing, supervision, and regular updates.

### Data analysis

For the statistical processing of our data, we will employ two software tools: the Statistical Package for the Social Sciences (SPSS) version 27.0 from IBM and R version 4.0.5 from The R Foundation for Statistical Computing. Our analysis will begin with data cleaning to ensure accuracy and reliability. We will evaluate the perceived importance of vaccination through responses to the question, "How important do you think vaccines are for your child’s health?" with options ranging from "Not at all important" to "Very important." Categorical data, such as these responses, will be summarized using frequency counts and percentages. In contrast, continuous variables will be expressed in terms of their means and standard deviations (SD). Statistical testing for differences between groups will be conducted using chi-square tests for categorical variables and t-tests or analyses of variance (ANOVA) for continuous variables. To identify significant predictors of HPV vaccination uptake, we will construct three logistic regression models in R, utilizing the generalized linear model function in the Finalfit 1.0.4 package. The first model will incorporate sociodemographic variables, the second will include variables related to HPV vaccination derived from the BeSD tool, and the third model will integrate predictors that show a strong correlation with vaccine relevance as identified in the first two models. In handling missing data, we will apply listwise deletion, a method where any record with missing data is excluded from the analysis. This approach assumes that the missing data are missing completely at random. For all statistical tests, a p-value threshold of 0.05 will be used to determine statistical significance, ensuring rigor in identifying meaningful associations in our study.

### Phase 2: Qualitative approach

By adhering to the COREQ (Consolidated Criteria for Reporting Qualitative Research) guidelines, this study will provide a comprehensive and detailed framework, ensuring methodological rigor and clarity in examining the contextual data on the perceptions and experiences of caregivers and healthcare workers regarding the drivers of HPV vaccination.

### Research team and reflexivity

Our research team comprises experienced professionals in public health, epidemiology, and social sciences, each bringing a wealth of knowledge and a unique perspective to the study. The team’s diverse expertise is crucial in ensuring a comprehensive approach to qualitative research, particularly in understanding health behaviors and vaccination uptake. There is no prior relationship between the research team and the study participants, ensuring an unbiased approach to data collection and interpretation. The team’s reflexivity, or awareness of their own biases and the potential influence on the research process, will be continuously monitored and discussed throughout the study to maintain objectivity and rigor in qualitative analysis.

### Participant selection and saturation point

In selecting participants, maximum variation purposive sampling will be employed to ensure a rich and diverse collection of perspectives on HPV vaccination [17,18]. This method involves deliberately choosing individuals who are especially knowledgeable about or experienced with the phenomenon of interest. In our study, this will include caregivers of children aged 9 to 14 years and frontline healthcare workers in the eThekwini District. The purposive sampling approach is critical in qualitative research for its effectiveness in gathering in-depth, meaningful data from participants who can provide significant insights into the study’s objectives. The selection process will aim to include a wide range of demographics, socio-economic backgrounds, and experiences to ensure a comprehensive understanding of the drivers of HPV vaccination.

The sample size for qualitative studies is typically determined by the point of data saturation, which is reached when new data no longer contributes to a better understanding of the studied phenomenon, but instead repeats what has already been expressed [19]. The point of saturation cannot be predicted in advance, so we have planned to recruit 8–10 minimum caregivers, as well as 20 healthcare workers in accordance with the usual points of data saturation reported in qualitative studies [20]. While our initial participant estimates are based on typical saturation points in similar studies, we are prepared to adjust our sample size if necessary, continuing recruitment until saturation is achieved to ensure a comprehensive understanding of the drivers of HPV vaccine uptake [21].

### Setting

See above. The study will take advantage of the varied demographic and socio-cultural landscape of the district, thereby enriching the study’s findings with contextually rich and nuanced data.

### Data collection

Data collection will comprise two primary methods: semi-structured in-depth interviews and focus group discussions. Around 40participants will engage in in-depth interviews, offering detailed, personal accounts of their experiences and perceptions regarding HPV vaccination [22]. Additionally, 10 participants will take part in each focus group discussion, providing a platform for group dynamics and collective views to emerge, fostering a deeper understanding of shared experiences and societal influences on vaccination decisions [22]. The interviews and FGDs will be conducted in the native Zulu language to ensure comfort and clarity for the participants. Each session is planned to last between 20 to 30 minutes and will be audio-recorded using dual recording devices to ensure data reliability. This mixed method approach allows for a comprehensive exploration of the individual and collective factors influencing HPV vaccine uptake.

We have selected a combination of in-depth interviews and focus group discussions for our study because this mixed method approach enables a more comprehensive comparison of perspectives, enhances the completeness of data, and improves the overall trustworthiness by ensuring confirmability, dependability, crefdibility, and transferability of findings [23]. The focus groups and interviews will be conducted across various communities by eight specially trained research assistants, with two assigned to each area. These assistants will actively engage with the communities, distributing informational pamphlets at local schools, clinics, and hospitals to inform potential participants about the study. Individuals expressing interest will be invited to participate, and appointments will be scheduled accordingly. To facilitate these sessions, we have developed interview guides, included as Appendices 2 and 3, which are structured to elicit rich, detailed responses. These guides are designed with open-ended questions, allowing participants to share their experiences and perspectives freely, thus providing depth and context to their responses. These qualitative interview guides will be used both for the individual in-depth interviews and for the focus group discussions, ensuring consistency in the data collection process across different methodologies.

### Data analysis and reporting

All recordings from focus groups and interviews will be verbatim transcribed, while omitting all personal identifiers. The analysis process will be dynamic and iterative, employing NVivo software for efficient data management and thematic analysis. The initial coding of transcripts (first 10) will be undertaken independently by two researchers to establish a preliminary coding framework. This framework will then be refined through regular team discussions, ensuring that the emerging themes are comprehensive and accurately reflect the data. These discussions will also serve to continually assess and refine the coding process, ensuring that the themes developed are robust and representative of the data. This collaborative approach to analysis is pivotal in qualitative research, as it allows for multiple perspectives to be considered, enhancing the depth and breadth of the analysis. The final themes will provide a detailed mapping of the behavioral and social drivers of HPV vaccination uptake in the district, offering valuable insights for public health interventions.

### Expected outcome

The expected outcome of this qualitative phase is a set of well-defined themes that encapsulate the perceptions and understandings of caregivers and healthcare workers regarding HPV vaccination. These themes are anticipated to offer profound insights into the various factors that influence HPV vaccine uptake, ranging from individual beliefs and knowledge to broader social and cultural influences. The richness of these findings is expected to contribute significantly to the field of public health, particularly in understanding vaccination behaviors. These insights could be instrumental in shaping future vaccination campaigns and policies, not only in the eThekwini District but also in similar contexts, thereby having a far-reaching impact on public health initiatives and vaccine uptake strategies.

### Ethical considerations

Ethical approval has been obtained from the University of Cape Town Faculty of Health Sciences Human Research Ethics Committee (HREC Reference: 286/2021). We have also obtained permission from the KwaZulu-Natal Provincial Department of Health.

The study process will comply with the requirements of the latest version of the Declaration of Helsinki (7^th^ revision, 2013). Verbal and written information about the study will be provided to all participants taking part in interviews (Appendix 4 in S1 File). Written informed consent will be obtained from all research participants before proceeding with the research (Appendix 5 in S1 File). The consent form will make explicit the following aspects: the voluntary nature of participation, that there will be no negative consequences if they decide not to participate and that they will be asked explicitly for permission for the interview to be digitally recorded and that this is also voluntary. Written consent will be obtained from all research participants before proceeding with interviews or focus groups. With permission of participants, interviews and focus groups will be audio-taped.

Details from interviews will be entered into a study-specific database on the day of collection (stakeholder group, participant ID etc.). Study data, including audio-recordings, will be stored on password-protected computers and shared with the study team only. All tape recordings on the digital recorders will be destroyed following safe storage and transcription, and identifying information will be removed from all transcripts. Reports of the findings will not identify individual participants. Participant anonymity and confidentiality will thus be ensured.

### Confidentiality

Participants must agree to abide by Chatham House Rule during focus groups. This rule states that ’when a meeting, or part thereof, is held under the *Chatham House Rule*, participants are free to use the information received, but neither the identity nor the affiliation of the speaker(s), nor that of any other participant, may be revealed. The application of this rule will protect participants who may wish to present or discuss controversial material or outline challenging circumstances.

### Dissemination of findings

The report of the main study findings will be shared with eThekwini District officials and stakeholders who took part in interviews and focus groups. The findings will also be communicated through academic publications and meetings.

## Discussion

### Strengths and limitations

The convergent parrallel mixed method design employed in this study, integrating both quantitative and qualitative approaches, offers a comprehensive understanding of the behavioral and social drivers of HPV vaccination in the eThekwini District. The strength of this design lies in its ability to triangulate data, thereby enhancing the validity and depth of the findings. Quantitative data provides a broad overview of the vaccination landscape, identifying general trends and patterns, while qualitative insights delve into the nuanced experiences and perceptions of caregivers and healthcare workers. This holistic approach allows for a more complete and contextually rich understanding of the factors influencing HPV vaccine uptake, which is crucial for developing effective interventions. However, this study is not without limitations. The mixed method design, while robust, can be resource-intensive in terms of time and logistics. Additionally, the interpretation of findings requires careful consideration to avoid potential biases inherent in combining data from different methodologies. These limitations notwithstanding, the mixed method approach is instrumental in capturing a multi-dimensional view of vaccination behaviors, crucial for informed decision-making in public health.

### Impact on policy and practice

The findings of this study have significant implications for both policy and practice. Understanding the various factors that influence HPV vaccine uptake is crucial for public health authorities and policymakers in developing targeted strategies to increase vaccination coverage. By identifying specific barriers and facilitators to HPV vaccination, this research can inform the development of tailored communication campaigns, educational programs, and policy initiatives that address the unique needs and concerns of the eThekwini District’s population. Moreover, the insights gained can guide healthcare providers in adopting more effective practices for vaccine promotion and administration. Ultimately, by improving HPV vaccine coverage, these informed interventions contribute to the broader goal of cervical cancer prevention. This research underscores the importance of context-specific, evidence-based approaches in public health planning and highlights the potential of such studies to effect meaningful change in health outcomes at both the local and national levels.

## Supporting information

S1 File (DOCX)

## Abbreviations

UCT: University of Cape Town
SAMRC: South African Medical Research Council
HPV: Human papillomavirus
WHO: World Health Organisation
BeSD: Behavioural and social drivers
