# Supplementary material for: Behavioural and social drivers of human papillomavirus vaccination in eThekwini District of KwaZulu-Natal Province, South Africa
Source: PLoS One. 2024 Dec 31;19(12):e0311509. doi: 10.1371/journal.pone.0311509 (PMC11687660; doi:10.1371/journal.pone.0311509)
Supplement: S1 File — (DOCX) [file pone.0311509.s001.docx]

#

**Supporting Information**

**Appendices 1 to 5**

# Appendix 1: Adapted HPV Vaccination Survey Questionnaire

**Survey –Screening**

Good [morning / afternoon]. I am [interviewer's name] with the University of Cape Town and the South African Medical Research Council(SAMRC).We would like to have a conversation with you with regards to the vaccine against human papillomavirus(HPV). HPV is a type of virus that causes infection and cancer of the cervix in women. We are interviewing parents from eThekwini district in KwaZulu-Natal to help improve the HPV vaccination services in our country.

***If potential participant informs you that they do not have children, end the interview.***

I know you are busy, so this will take only 30 minutes. Your participation is completely voluntary and anonymous. If you do not want to answer a question or wish to stop the interview, just let me know.

1. Would you be willing to take the survey?

- Yes
- No

If "yes" to Q1: Thank you very much. Do you have any questions for me before we begin? ***Proceed to survey screener after addressing any questions.***

***If "No" to Q1****:* ***Thank you very much****.* ***End interview****.*

2. What is your gender?

- Man
- Woman
- Non-binary or transgender
- Prefer not to say

3. In what year were you born? (CCYY)

***(4-digit figure) if born in 2005 or later thank participant and end interview***.

4. What is the highest educational level that you have obtained?

- No formal education
- Primary
- Secondary
- Matric
- Tertiary
- Other

5. Which of the following best describes your total monthly household income (from all sources) at this time

- No Income
- Between R0 and R 2 999 per month
- Between R3 000 and R10 000 per month
- Between R10 000 and R20 000 per month
- Between R20 000 and R30 000 per month
- R30 000 or more per month

6. To your knowledge, were you vaccinated as a child?

- Yes
- No

7. Have you ever been vaccinated for flu?

- Yes
- No

8. Are you vaccinated or planning to vaccinate against COVID-19?

- Yes
- No
- I don't know
- I don't want the vaccine

9. Are you the parent or are you responsible for any child who is 8 years old or older?

- Yes
- No

***If "Yes" to Q9, participant is eligible for survey, continue to Q10.***

***If "No" to Q9, participant is not eligible for survey, and you can thank the participant as follows:***

Thank you for answering those questions. Unfortunately, you are not eligible to participate in the survey since we are going to ask questions about children and you are currently not having children. We would like to thank you very much for taking the time to answer my questions. ***End Interview***.

10. How many children do you have who are 8 years old or older?

*(****Record number of children****) ##*

The next questions are about you and about your **youngest** child who is 8 years old or older.

11. What is your relationship to the child? Are you the…

- Mother
- Father
- Grandparent
- Uncle or Aunt
- Brother or Sister
- Other? [IF “OTHER”: Please specify ______________________]

12. How old is your child?

- _____ years [***Record age in number of completed*** ***years***].

13. Is your child a……?

- Boy
- Girl
- Non-binary or transgender
- Prefer not to say

**Survey – Main Survey Items**

14. How important do you think vaccines are for your child's health? Would you say…

- Not at all important
- A little important
- Moderately important
- Very important

15.How much do you think vaccinating children protects other people in your community from diseases? Would you say other people are…

- Not at all protected,
- A little protected,
- Moderately protected
- Very protected

16.Do your religious or spiritual beliefs **encourage** vaccinating your child, **discourage** vaccinating you child, or would you say this **doesn't apply** to you?

- **Encourage** vaccinating
- **Discourage** vaccinating,
- would you say this doesn't apply to you?

17. Have you ever heard of human papillomavirus or HPV?

- Yes
- No

18. Where have you heard about HPV before? Mark all that apply

- Doctor, nurse or other health care professional
- Family or friends
- Newspaper or magazine
- Television
- Internet
- Radio
- Don't remember
- Other [Please specify]

19. Did you know that HPV can cause cervical cancer and many other cancers? Yes

- No

20. Are you aware that all grade five girls in public schools are being offered the HPV vaccine?

□ Yes

□ No

If YES, how did you hear about it? (Tick all that apply)

- from family or friends
- On Radio
- from Education Officials
- On Television
- from Health Officials
- Other (specify):

21. Would you allow your daughter or a close relative to get the HPV vaccine?

- Yes
- I don't know
- No
- She has already taken it

If your answer is NO, please indicate why?

- I am against all vaccinations
- The vaccine is not safe
- The HPV vaccine will make young girls start sexual activity early
- My religion does not allow vaccination
- The HPV vaccine is not necessary
- Other (specify):

22. How safe do you think the HPV vaccine is for your child? Would you say…

- Not at all safe
- A little safe
- Moderately safe
- Very safe

23. How concerned are you that HPV vaccine could cause your child to have a serious reaction? Would you say...

- Not at all concerned
- A little concerned
- Moderately concerned
- Very concerned

24. Do you think that the HPV vaccine should be offered to boys as well?

- Yes
- I don't know
- No

25. Which of these delivery systems do you think are appropriate for HPV vaccine delivery (select all that apply)?

- Schools
- Health facilities/clinics
- Community (village market, churches)
- Other (Specify):

#

# Appendix 2: Adapted Qualitative Interview Guide for Parents or Caregivers

**GENERAL**

1. **Would you like to start off by telling me a little about yourself?**

**SOCIAL PROCESSES & NORMS**

1. **Let's talk a little bit about your decision to get/not to get the human papillomavirus (or HPV) vaccine. How did you go about making this decision?**

*Probes*

- *Was there anyone else involved in the decision? Did you discuss your decision with anyone? (prompt spouse, family, friends, community members?).*
- *Was your child(ren) involved at all in the decision? Did you talk to her about your decision? Did she agree/disagree with your decision. Why/why not?*

1. **If the HPV vaccine is recommended by healthcare workers, would you trust their advice?**

*Probes:*

- *What about family and friends: If the HPV vaccine is recommended by family and friends, would you trust their advice?*
- *What about religious or community leaders: If the HPV vaccine is recommended by religious or community leaders, would you trust their advice?*

**PRACTICAL FACTORS**

**If the participant's child(ren) has received the HPV vaccine, ask question #4. If the participant's child(ren) has NOT received the vaccine skip question #4.**

1. **I'd like to hear about how your child received the vaccine.**

*Probes*

- *Where did they get the vaccine?*
- *Did you give permission for your child(ren) to get the vaccine? How did this work?*
- *Who gave them the vaccine?*
- *Do you know how many doses they received?*
- *How did you feel about the process? Was there anything you liked? Was there anything you disliked?*
- *Did your child(ren) have any thoughts or feelings about the process?*

1. **Currently the HPV vaccination programme is only delivered in public schools. What do you think about this approach?**

*Probe*

- *Is there anything you like about this approach? Is their anything you don't like about this approach?*

1. **Currently the HPV vaccination programme in schools is only given to girls. What are your thoughts about this?**

*Probes*

- *Should the programme be extended to boys? Why/why not?*

1. **Currently the HPV vaccination programme is not available at public health facilities. What are your thoughts about this?**

*Probe*

- *Do you think HPV vaccination should be made available at public health facilities? Why/why not?*

**THINKING AND FEELING**

1. **Can you tell me what you've heard about the HPV vaccine?**

*Probes:*

- *Do you know why the vaccine is given? Have you heard anything about the benefits of the vaccine?*
- *Have you heard anything that worries you about the vaccine? (e.g. safety, side-effects?)*
- *Have you heard anything that makes you feel positive/good about the HPV vaccine?*

1. **HPV is one of the most common viral infection of the private parts. And certain types of HPV infections can cause cervical cancer and other kinds of diseases in both men and women. The HPV vaccine is therefore given to help prevent HPV infection and the diseases it causes. How worried are you about your child getting the cervical cancer?**

*Probes:*

- *Why do you feel that way?*
- *How likely do you think it is that they would get the HPV infection?*
- *How worried are you about your child getting cervical cancer? Do you know that about 85% of estimated female cancer deaths occur in African countries?*

1. **How do you feel about your child(ren) getting the HPV vaccine?**

*Probes:*

- *Alignment with spiritual or religious beliefs? Other beliefs?*
- *Thoughts about the safety of the vaccine? What about side-effects?*
- *Thoughts on whether it will work?*

**MOTIVATION AND INTENTION**

1. **Has your child(ren) received the HPV vaccine?**

*Probe:*

- *Can you tell me why/why not?*

1. **Has your child(ren) received any other vaccines?**

*Probe:*

- *Can you tell me why/why not?*

**SOURCES OF INFORMATION AND TRUST**

1. **Have you received any information about the HPV vaccine from your healthcare provider?**

*Probes:*

- **If yes:** *Did you trust this information? What makes you feel that way?*
- **If no:** *Would you trust information given to you about the HPV vaccine by your health care provider? What makes you feel that way?*

1. **Have you received any information about the HPV vaccine from your child(ren) 's school/teachers?**

*Probes:*

- **If yes:** *Did you trust this information? What makes you feel that way?*
- **If no:** *Would you trust information given to you about the HPV vaccine by your child(ren) 's teacher/school? What makes you feel that way?*

1. **Have you received any information about the HPV vaccine from any other sources (e.g. the internet, friends, family member, media or other sources?)**

*Probes:*

- **If yes:** *Did you trust the information about the HPV vaccine given to you by [the sources participant mentions]? What makes you feel that way?*
- **If no:** What sources of information about the HPV vaccine would you trust?

1. **Is there anything else you would like to add or comment on?**
2. **Any questions at this stage?**

**Thank you so much for your time!**

Appendix 3: Adapted Qualitative Interview Guide For Frontline Healthcare Workers

**GENERAL**

1. **Is your role at the health facility where you work primarily administrative,medical, or the other?**
2. **Are you involved with HPV vaccination?**

*Probe*

- *Can you tell me more about this involvement? Do you administer HPV vaccines? To whom? Where do you administer them? How long have you been doing this work?*

**SOCIAL PROCESSES & NORMS**

1. **Who do you think is involved in the decision-making for HPV vaccination?**

*Probes*

- *Mother, Father, other family, friends, community members, religious or community leaders, teachers, healthcare workers?*
- *Do you think the daughter herself is involved in the decision about whether she will get the HPV vaccine?*

**PRACTICAL FACTORS**

1. **I'd like to hear your thoughts and experiences with the HPV vaccination programme in schools?**

*Probes*

- *What do you think is working well?*
- *What do you think is not working well? E.g. are there any challenges with scheduling of visits and/or access to schools? Are there any issues associated with determining eligibility and/or the consent process? Were there any issues associated with giving the vaccine itself?*
- *Have you received any feedback about the programme from parents?*
- *What do you think could be done to address these challenges you've mentioned?*

1. **Currently the HPV vaccination programme in schools delivers 2 doses of the vaccine per year. Evidence suggests that there is a significant drop in uptake from the 1^st^ to the 2^nd^ dose. Why do you think this might be so?**
2. **Currently the HPV vaccination programme does not include private schools and the HPV vaccine is not available at public health facilities. What are your thoughts about this?**

*Probes*

- Should the programme be extended to private schools? Why/why not?
- Should the HPV vaccine be made available at public health facilities? Why/why not?

1. **Currently the HPV vaccination programme in schools is only given to girls. What are your thoughts about this?**

*Probes*

- Should the programme be extended to boys? Why/why not?

**THINKING AND FEELING**

1. **To what extent do you think parents and girls think that the HPV vaccine is necessary?**

*Probe*

- *Do you think they feel concerned that their child could get the HPV infection? Cervical cancer? Why/why not?*

1. **Do you think parents and girls have any worries or concerns about the HPV vaccine?**

*Probes*

- *E.g. side effects? Adverse effects? Will promote sexual activity? Dis-alignment with spiritual or religious beliefs or other beliefs?*

**MOTIVATION AND INTENTION**

1. **Would you encourage and support young girls to be vaccinated?**

*Probes:*

- *Why/why not?*

1. **To what extent do you think parents are willing to have their daughters vaccinated?**

*Probes*

- *Why/why not?*
- *What about the daughters- from your experience, to what extent are they willing to be vaccinated? Why/why not?*

**SOURCES OF INFORMATION AND TRUST**

1. **Do you think parents and girls know enough about the HPV vaccine?**

*Probes:*

- *Do you think they know about the reasons for/benefits of the vaccine?*
- *Where do you think parents and girls receive information about the HPV vaccine (Probes e.g. the internet, friends, family member, media, religious or community leaders, teachers/schools, healthcare workers/the clinic, other sources?*
- *What sources of information about HPV vaccination do you think parents and girls trust? (Probes e.g. the internet, friends, family member, media, religious or community leaders, teachers/schools, healthcare workers/the clinic, other sources?)*

1. **Are there any other factors, that we haven't already discussed, that you think might make some parents or girls unwilling to be vaccinated?**
2. **What do you think could be done to encourage parents and girls to accept the HPV vaccine?**
3. **Is there anything else you would like to add or comment on?**
4. **Any questions at this stage?**

**Thank you so much for your time!**

Appendix 4: Information Leaflet

**Title of the study:** Behavioural and social drivers of human papillomavirus vaccination in eThekwini District of KwaZulu-Natal Province

Dear Participant

We would like to invite you to participate in a research study to understand reasons as to why so many eThekwini district adolescents do not receive the HPV vaccine they require.

**Background:** Immunization is undisputedly one of the most cost-effective public health interventions. Despite the unparalleled success that immunization has made in the control of vaccine preventable diseases, immunization coverage is suboptimal in South Africa. An assessment of the performance of the programme reveals high coverage of more than 80% of eligible adolescents reached with at least the first dose of the vaccine. However, there is wide variation in coverage between and within districts; with the lowest coverage being 40%, registered in a sub-district in KwaZulu-Natal Province

The aim of this research is to investigate barriers to optimal uptake of HPV vaccination services in eThekwini district of KwaZulu-Natal province and to develop contextualized strategies to increase the HPV vaccination coverage.

You are invited to participate in this research because you have been identified as someone who may have important information on why so many eThekwini district children do not access life-saving HPV vaccines.

**Your Rights**

Should you agree to participate in this study, you will be asked to participate in an interview, lasting no more than an hour. The interviewer will ask about your perspectives of barriers to HPV vaccines uptake, and ways that these barriers might be addressed. Your answers will be audio taped. This ensures that valuable information from this interview is not missed. The information on the tape will be transcribed for analysis purposes.

You will not be identified in any way. Although we ask you to sign a consent form to participate, this will be held securely and separately from the research data. The data collection forms will not record participant names or any identifying details. The information obtained from this interview will be treated with strict confidentiality. Your name will NOT appear on the transcription. Members of the research team will undertake the analysis of the interviews. Your audio recording will not be released to any persons or entities other than the research team. The audio recording and typed transcription of the interview will be stored in a password protected computer file to which only the research team staff will have access. The anonymous scientific data – in which no individuals will be named or identified – may be presented at meetings, and published in national or international journals, for dissemination purposes.

Your participation in this study is completely voluntary, and choosing to, or not to, participate will have no repercussions. You are free to withdraw from the interview at any time, or decline to answer any of the questions without penalty.

You will receive a reimbursement of R100 for taking part in this research study.

Yours sincerely

Dr Muki Shey, **Principal Investigator:**

**Phone: 021 650 1731; email: muki.shey@uct.ac.za**

Phelele Bhengu, **Student researcher** Phone: 0818419073

Email: bhnphe001@myuct.ac.za

#

# Appendix 5: Informed Consent Form

**Title of the study:** Behavioural and social drivers of human papillomavirus vaccination in eThekwini district of KwaZulu-Natal Province

**Why is this study being done?** The aim of this research is to investigate barriers to optimal uptake of HPV vaccination services in eThekwini district of KwaZulu-Natal province in South Africa and develop contextualized strategies to increase the vaccination coverage.

**Why are you being asked to take part?** You are invited to participate in this research because you have been identified as someone who may have important information on why so many eThekwini district children do not access life-saving HPV vaccines.

**How many people will take part in the study?** At least 880 participants will take part in the study.

**Will the results of this study be shared with you?** The results of this study will be shared with participants at presentations or if individually requested.

**Will any of your information or data be stored and used for research in the future?** We will request your permission to store information collected from you in an anonymous manner.

**Will your information be shared with other researchers?** The information may be shared with other researchers to assist with future research. This information will not be sold. All future studies using the data will need permission from the UCT Faculty of Health Sciences Human Research Ethics Committee first. Your name will never be revealed even if we share your data.

**Are there any benefits to you for being in the study?** Taking part in the study will not benefit you directly, but the study will help us understand barriers to optimal uptake of HPV vaccination services in eThekwini district.

**What are the risks and discomforts of this study?** There is no direct risk involved in participating in this study. Information collected from you will be kept confidential and only accessible to research staff.

**What happens at the end of the study?** Information collected will be stored, if permission is given, for further analyses as need arises. The results from this study will be presented at local or international conferences.

**Who will see the information which is collected about you during the study?** Questionnaires will be marked with a number and not your name to keep your identity and the information you provide private.

**Will you receive any reward for taking part in this study?** You will not be paid for allowing the researchers to collect information from you.

**What other choices do you have?** You can decide not to take part in the study. Even if you give your permission now, you can still change your mind later. Your decision will not affect future relations with the study team or the University of Cape Town. Your decision will also not affect any aspect of your current employment situation or access to healthcare services for your family.

**Who do I speak to (or contact) if I have any questions about the study?** You may ask any questions you have now, and for future questions, you are encouraged to contact: Dr Muki Shey on phone number **021 650 1731** as the principal investigator or Miss Phelele Bhengu on 0818419073 as lead student researcher. In case of any questions regarding the welfare and rights of research participants, you should contact the UCT Faculty of Health Sciences Human Research Ethics Committee on phone number **021 406 6492**

**If you would like to participate, please indicate your choice(s) below:**

I agree to the participate in this study voluntarily

I consent that my data can be stored and used for future research

I consent for my data to be used for this research ONLY and NOT any future research

Signed at (*place*) ......................…........…………….. On (*date*) …………....……….. 2021

Participant name: ..................................... ………………………

Signature of participant: …………………………………………..

***Audio taping:* I understand and agree that the interview will be audio recorded**

Signature of participant: …………………………………………..

Investigator name: ................................... ………………………

# Signature of investigator: …………………………………………..
